# Patellar tendinopathy caused by a para-articular/extraskeletal osteochondroma in the lateral infrapatellar region of the knee: a case report

**DOI:** 10.1186/1757-1626-2-9341

**Published:** 2009-12-17

**Authors:** Kutay Engin Ozturan, Istemi Yucel, Husamettin Cakici, Melih Guven, Kamil Gurel, Sergulen Dervisoglu

**Affiliations:** 1 Department of Orthopaedics and Traumatology, Abant Izzet Baysal University, Izzet Baysal Medical Faculty, Turkey; 2 Department of Orthopaedics and Traumatology, Duzce University, Duzce Medical Faculty, Turkey; 3 Department of Radiology, Abant Izzet Baysal University, Izzet Baysal Medical Faculty, Turkey; 4 Department of Pathology, Istanbul University, Cerrahpasa Medical Faculty, Turkey

## Abstract

Patellar tendinopathy is characterized by activity-related anterior knee pain. It is most commonly related to sports activity, but has also been reported in the non-athletic population. Most injuries are caused by microtrauma, resulting in tendinitis or tendinosis. Extraskeletal paraarticular osteochondromas, which occur in the soft tissues near the joint, are rare. The infrapatellar fat pad and joint capsule are the most common sites of these tumors. Here, a case of patellar tendinitis caused by an extraskeletal paraarticular osteochondroma is reported. The symptoms included intensifying pain upon flexion and a palpable click that was located at the medial side of the mass. The patient was pain-free within 3 weeks after excision of the tumor and the clicking disappeared. To our best knowledge, no other case of patellar tendinitis caused by an extraskeletal paraarticular osteochondroma has been reported in the English literature.

## Introduction

Patellar tendinopathy (PT) affecting the patellar or tibial insertion of the patellar tendon is one of the most common causes of anterior knee pain. Chronic overload resulting in microtrauma is the primary etiological factor in this pathology [1].

Extraskeletal paraarticular osteochondromas (ESPAOCs) are rare benign bone tumors that typically appear in the infrapatellar region. These tumors arise from the juxtaarticular soft tissue and may show histological features suggestive of a malignant process [2]. Accurate diagnosis of these cases is important to prevent unnecessary aggressive surgery [3]. We treated a patient with PT at the mid-portion of the patellar tendon that was caused by compression/impingement of an ESPAOC. To our best knowledge, no other such case has been reported in the English literature.

## Case presentation

A 60-year-old Turkish female presented with a swelling in the anterolateral region of the left knee. The swelling had been present for 10 years, and had been increasing gradually in size over the previous 3 years. Two years prior to presentation, the patient began to experience pain on the medial side of the swelling, which intensified upon flexing the knee.

A palpable 5 × 4.2 × 3.5 cm hard bony mass was detected in the lateral infrapatellar area. The mass was partially mobile in the long-axis direction, but fixed with regard to lateral (side-to-side) motion. The range of motion was 130° and a click on the medial side of the mass was palpable at 70° of flexion.

**Figure 1:**
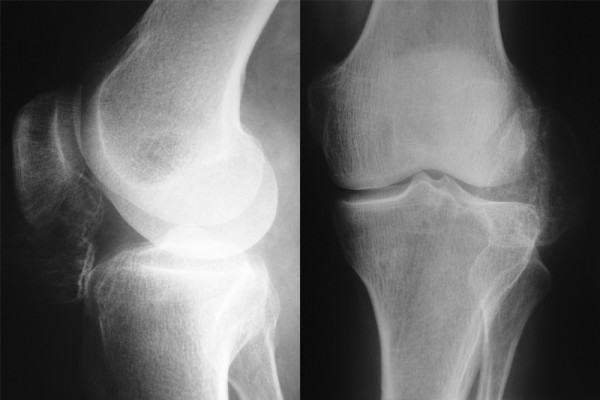
**A and B.** The osteochondral mass adjacent to patella in the left knee is seen at AP and lateral radiographs.

**Figure 2:**
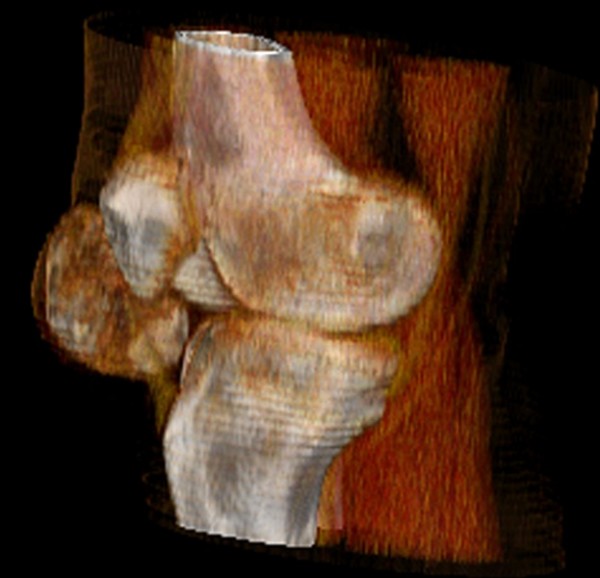
**Volume rendering reformatted CT image shows the position of the osteochondral lesion with respect to patella, tibia and patellar tendon**. The mass is not attached to adjacent bones.

**Figure 3:**
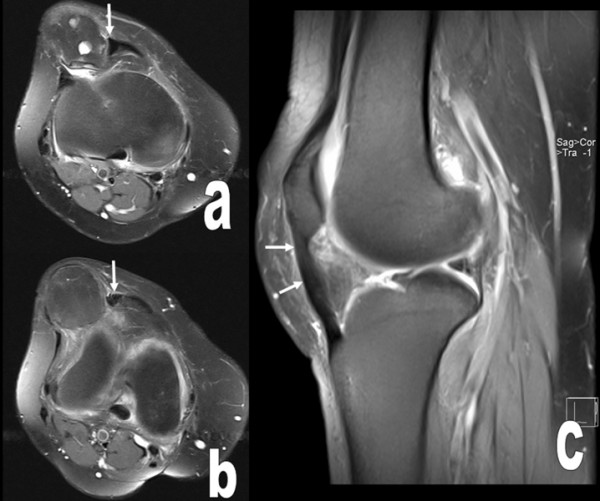
**The osteochondral mass within the retinaculum, its compression to the patellar tendon, increased thickness and signal of the patellar tendon (arrows) are seen at axial fat saturated proton density (a, b) and sagittal fat saturated T2 weighted MR images (arrows) (c)**.

Radiographs revealed a large, well-circumscribed, mineralized mass inferolateral to the patella (Figure 1a, and Figure 1b). Computed tomography (CT) was performed to examine the relationship of the mass to the patella, femur, and tibia. There was no continuity between the mass and these bones (Figure 2). Magnetic resonance imaging (MRI) showed the mass within the retinaculum. Compression of the patellar tendon and increased thickness and signal of the patellar tendon were observed in axial fat-saturated proton density and sagittal fat-saturated T2-weighted MRI images (Figure 3a, 3b, 3c).

**Figure 4:**
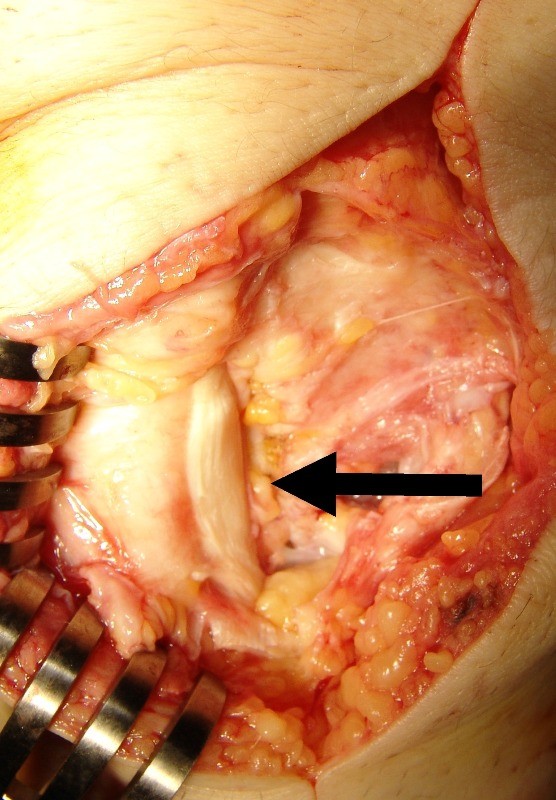
**Lateral side of the patellar tendon was contoured due to compression by the oval mass and tendon thickening near to the tumor was shown after removal of the tumor**.

The lesion was excised through a longitudinal anterolateral incision. Intraoperatively, the lesion was found to be separate from the patella and completely extraskeletal and extraarticular. During surgery, patellar tendon thickening near to the tumor was observed and lateral side of the tendon was contoured due to compression by the oval mass (Figure 4).

**Figure 5:**
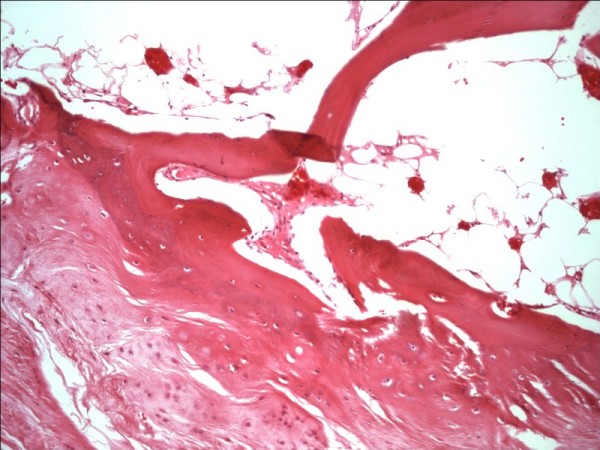
**Well-circumscribed mass which shows hyaline cartilage matures into underlying trabecular bone**. Fatty bone marrow is seen at the intertrabecular space. HE×100.

**Figure 6:**
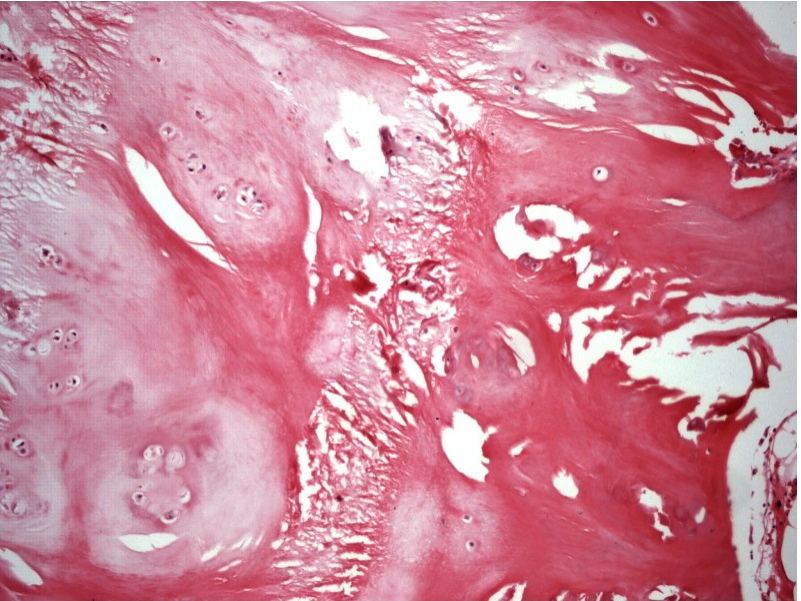
**Hyaline cartilage with clone forming chondrocytes matures into bone**. HE×100.

The resected specimen was a lobulated mass with a smooth fibrous outer surface. It measured 5 × 4.2 × 3.5 cm and the cut surface revealed approximately 0.2 cm thick, translucent, bluish cartilage at the periphery that merged into spongious bone and fatty marrow at the center (Figure 5). Microscopically, osteochondromatous architecture was observed, which consisted of chondral ossification from the periphery to the center. Chondrocytes in the cartilage zone had small dark-stained nuclei that lacked cytologic atypia. Small clone formation of chondrocytes and fatty bone marrow between the bone trabeculae were observed (Figure 6).

Range of motion exercises began on postoperative day 1. The patient was completely pain-free within 3 weeks postoperatively and the clicking disappeared. Twelve months after surgery, there was no recurrence of the tumor and the patient was asymptomatic.

## Discussion

PT is characterized by activity-related anterior knee pain, focal patellar tendon tenderness, and intratendinous imaging changes [4]. PT is usually diagnosed based on clinical symptoms alone, although MRI and Doppler ultrasonography may also provide diagnostic insight. PT is most commonly related to sports activities, but has also been reported in the non-athletic population [5].

Histopathological studies have consistently shown that the pathology underlying chronic PT is degenerative (tendinosis) rather than inflammatory (tendinitis) [4,6]. Tendinosis is defined as widening of the tendon, disturbed collagen distribution, revascularization, and increased cellularity histopathologically [7,8]. PT is most commonly localized at the patellar insertion of the tendon, whereas the tibial insertion is rarely affected. Although the etiology of PT is unknown, impact loading, genetic make-up, and inefficient lower limb biomechanics are thought to contribute to this condition [9]. Sudden maximal muscle-tendon unit exertion (e.g., jumping) is the greatest risk factor [1]. In our case, chronic compression/impingement resulting from an ESPAOC, which was partially mobile in the longitudinal direction, was thought to be an etiological factor. The PT was localized at the mid-portion of the patellar tendon, in contrast to the classical localization of PT.

Osteochondroma is the most common benign bone tumor and usually occurs in the metaphyseal region of the long bones [10]. The vast majority of osteochondromas present as solitary lesions. The most common type of osteochondroma occurs in adolescents and children. These lesions usually have a pedunculated attachment to bone and grow away from the joint. In contrast, ESPAOC are rarely seen. These tumors appear in the soft tissues near to the joint. Infrapatellar fat pad and the joint capsule are the most common sites that the tumor originates and no bony attachment is observed [3]. The knee is the most frequently involved area [11]. Reith et al. [2] described the three main features of para articular osteochondroma, based on published cases: 1) the lesion presents as a single, dominant mass, both radiographically and grossly; 2) the mass is composed of bone and cartilage histopathologically, similar to typical osteochondromas; and 3) the mass is not intra articular. The differential diagnosis includes chondrosarcoma and synovial chondromatosis. A careful histopathological and radiological evaluation will distinguish ESPAOC from these tumors. The conventional osteochondromas stop enlarging after cessation of skeletal growth however ESPAOC's are more commonly seen to develop after skeletal growth has stopped [11].

Impingement symptoms or friction injuries related to osteochondromas are rarely reported. Onga et al [12] reported biceps tendinitis caused by an osteochondroma localized at the bicipital groove. Supraspinatus tendinitis related to distal clavicular osteochondromas has been reported by Ogawa et al [13] and Reichmister et al [14]. In our case, the pathology resulted from a similar mechanism: compression of the mass increasing with motion. However, in our case, the tumor was not continuous with the adjacent bone. Chronic compression/impingement of a partially mobile ESPAOC and the localization of the tumor were thought to be etiological factors of PT in this patient. In three previously reported cases, clinical symptoms disappeared shortly after excising the osteochondroma [12-14]. In our patient, clinical symptoms disappeared within 3 weeks after excising the tumor and no recurrence was observed within 12 months postoperatively.

## Conclusion

ESPAOC is a rare possible etiological factor in patellar tendinopathy. A careful radiological and histopathological work-up is important for making the correct diagnosis and preventing unnecessary aggressive surgery.

## Consent

Written informed consent was obtained from the patient for publication of this case report and accompanying images. A copy of the written consent is available for review by the Editor-in-Chief of this journal

## Competing interests

The authors declare that they have no competing interests.

## Authors' contributions

KEO operated the patient, collected data and prepared the manuscript, IY treated the patient and collected data, KG performed and analyzed the x-rays, CTs and MRIs, MG reviewed and corrected the manuscript, HC treated the patient, reviewed and corrected the manuscript, SD prepared, analyzed and interpreted pathological specimen. All authors read and approved the final manuscript.
